# Thyroid Disorders in Patients Treated with Dimethyl Fumarate for Multiple Sclerosis: A Retrospective Observational Study

**DOI:** 10.3390/antiox11051015

**Published:** 2022-05-21

**Authors:** Cédric O. Renaud, Panos G. Ziros, Amandine Mathias, Caroline Pot, Gerasimos P. Sykiotis

**Affiliations:** 1 Service of Endocrinology, Diabetology and Metabolism, Lausanne University Hospital and University of Lausanne, CH-1011 Lausanne, Switzerland; cedric.renaud@chuv.ch (C.O.R.); panos.ziros@chuv.ch (P.G.Z.); 2 Laboratories of Neuroimmunology, Neuroscience Research Center and Service of Neurology, Department of Clinical Neurosciences, Lausanne University Hospital and University of Lausanne, CH-1011 Lausanne, Switzerland; amandine.mathias@chuv.ch (A.M.); caroline.pot-kreis@chuv.ch (C.P.)

**Keywords:** Nrf2, Keap1, dimethyl fumarate, thyroid, Graves’ disease, goiter

## Abstract

Background: Dimethyl fumarate (DMF), a drug used for the treatment of multiple sclerosis (MS) and psoriasis, has been shown to activate the Keap1/Nrf2 antioxidant response. Nrf2 exerts pleiotropic roles in the thyroid gland; among others, single nucleotide polymorphisms (SNPs) in the gene encoding Nrf2 modulate the risk of Hashimoto’s thyroiditis (HT), suggesting that pharmacological activation of Nrf2 might also be protective. However, a patient with acute exacerbation of HT after starting DMF for MS was recently reported, raising questions about the thyroidal safety of Nrf2 activators. Methods: In a retrospective observational study, we investigated the prevalence and incidence of thyroid disorders (TD) among 163 patients with MS treated with DMF. Results: Only 7/163 patients (4.3%) were diagnosed with functional TD; most (5/163, 3.0%) were diagnosed before DMF treatment. Functional TD were diagnosed under or after DMF in only 2 patients (1.2%). Under DMF, one patient developed transient mild hypothyroidism with negative thyroid autoantibodies. After DMF discontinuation, another patient developed hyperthyroidism due to Graves’ disease. No patient developed thyroid structural disease under or after DMF. Conclusions: The very low incidence of functional TD indicates an overall very good thyroid tolerance of DMF, arguing against screening for TD in MS patients considered for or treated with DMF, and supporting the further study of Nrf2 activators for the prevention and treatment of TD.

## 1. Introduction

Multiple sclerosis (MS) is a complex immune-mediated disease of the central nervous system characterized by inflammation, demyelination, and axonal degeneration [1] that leads to chronic disability. Dimethyl fumarate (DMF) is a drug currently used as a disease-modifying agent for the treatment of relapsing-remitting MS as well as for the treatment of psoriasis. Its precise mechanism of action is still under study, and several possibilities have been proposed, including modulation of the immune system and stimulation of neuroprotective pathways, such as the endogenous antioxidant response system centered on the nuclear transcription factor nuclear factor-erythroid 2-related transcription factor 2 (Nrf2) [2,3]. Nrf2 regulates the basal and inducible expression of a battery of cell-protective genes [4]. In the absence of oxidative stress, Nrf2 binds to its cytoplasmic inhibitor Keap1 (Kelch-like ECH-associated protein 1) that targets Nrf2 for poly-ubiquitination and proteasomal degradation [5,6]. Oxidative stressors abolish the inhibition of Nrf2 by Keap1; Nrf2 then accumulates in the nucleus where it is transcriptionally active. Recent studies by our group in mice and humans showed that Nrf2 exerts pleiotropic roles in the thyroid gland [7,8]: it mediates antioxidant transcriptional responses and also directly stimulates the transcription of the gene encoding thyroglobulin (Tg), the precursor protein of thyroid hormones, while at the same time limiting Tg iodination, a critical step in thyroid hormone synthesis [9]. Single nucleotide polymorphisms (SNPs) in the gene encoding Nrf2 were found to modulate the risk of Hashimoto’s thyroiditis (HT) in conjunction with a previously identified HT-risk SNP in a selenoprotein cell-protective enzyme [10], thus providing evidence that Nrf2 has a protective role against autoimmune thyroiditis (AIT). Therefore, compounds that modulate Nrf2 activity might be promising candidates to treat or prevent AIT as well as other thyroid disorders (TD). In contrast to this hypothesis, a recent case report presented a 59-year-old patient with long-standing HT but normal thyroid function before DMF treatment, who developed subclinical hypothyroidism 2 months after starting DMF; this was attributed to acute exacerbation of her HT, with thyroid function normalizing within 2 months after DMF discontinuation [11]. This case report prompted us to investigate the prevalence and incidence of TD in patients with MS who receive treatment with DMF.

## 2. Patients and Methods

We conducted a retrospective observational study in patients with MS enrolled in the COOLIN’BRAIN cohort at the Service of Neurology of Lausanne University Hospital between 2005 and 2021. Patients of interest were those who had been treated or were still being treated with DMF as either first-line or subsequent therapy. The study was conducted in accordance with the principles of the Helsinki Declaration and the procedures followed were in accordance with institutional guidelines under protocols approved by the Ethics Committee of the Canton de Vaud (approval number CER-VD 2018-0622); all cohort participants had given written informed consent.

All patients fulfilled the criteria for MS at the time of diagnosis. In total, 163 patients with MS had received or were still receiving DMF. The electronic medical records of these patients were reviewed to document their thyroid gland status. Diagnosis of TD, either functional or structural, was retained if any of the following conditions were met: (i) TD was mentioned in an official out- or in-patient report; (ii) there was evidence of abnormal thyroid function tests (serum TSH, free T4 and free T3 levels were reviewed, whenever available) or elevated titers of thyroid autoantibodies against thyroperoxydase (anti-TPO), thyroglobulin (anti-Tg) or the TSH receptor (anti-TSHR); or (iii) there was evidence of abnormal findings in thyroid imaging studies. In the absence of these criteria, absence of TD was concluded. Of note, in the COOLIN’BRAIN cohort, thyroid functional and immunological status were not assessed regularly according to a standardized protocol, but either ad hoc for clinical suspicion of specific TD or as part of a general assessment for other clinical situations. The duration of DMF treatment and the follow-up time after DMF discontinuation were determined in relation to the date of the last visit recorded in the respective cohort’s database.

Biobanked serum samples of select patients who presented altered thyroid function during or after treatment with DMF or with a preexisting functional TD were analyzed further for thyroid hormonal and immunological status. Serum levels of TSH, free T4 and free T3 were measured by ECLIA (cobas e801, Roche Diagnostics, Basel, Switzerland), anti-TPO, and anti-TSHR antibodies by ECLIA (cobas e411, Roche Diagnostics, Basel, Switzerland) and anti-Tg antibodies by TRACE (Kryptor GOLD, ThermoFischer Scientific, Waltham, MA, USA) at Lausanne University Hospital.

Samples meeting the following criteria were selected for analysis: (i) the most recent sample before DMF initiation (pre-treatment sample); (ii) the most recent sample when the patient was still under treatment or the earliest pathological sample according to the medical record (during-treatment sample); and (iii) in patients no longer treated with DMF, the most recent sample after the end of treatment or the earliest pathological sample according to the medical record (post-treatment sample).

In our cohort, 73.0% of patients were female (*n* = 119/163); at the time of the study, about half of the total patients (*n* = 75/163, 46.0%) were still under DMF treatment. For the patients with ongoing DMF treatment, the treatment duration (mean ± standard deviation) was 27 ± 23 months. In patients who had discontinued DMF treatment, the treatment duration was 23 ± 19 months and the follow-up time after DMF discontinuation was 29 ± 20 months. DMF treatment was introduced at a median age (interquartile range) of 30 (31 to 48.25) years. Individual patient data are shown in Table S1 for the whole cohort and in Tables S2–S8 for select patients who are discussed further in the Results.

## 3. Results

### 3.1. Assessment of Thyroid Function Was Common in Patients with MS Treated with DMF

Less than one-tenth of patients (*n* = 14/163, 8.5%) had no recorded TSH value at all, whereas at least one TSH value was recorded before DMF treatment in the vast majority of patients (*n* = 109/163, 67.0%) and in more than one-third during DMF treatment (*n* = 60/163, 37.0%). Among patients who were no longer under DMF treatment (*n* = 88, 54.0%), 40.0% (*n* = 35/88) had at least one TSH value recorded after DMF discontinuation. Overall, about one-fifth (*n* = 29/163, 18.0%) of the patients had at least one TSH value recorded in all relevant periods (before and during treatment, and, in those who had discontinued treatment, after treatment as well).

### 3.2. Functional TD Were Rare among Patients with DMF Treatment

Only 7 of the total 163 patients (4.3%) were diagnosed with functional TD. In most of these patients (5/163, 3.0%), diagnosis had been made before DMF treatment: two female patients had been diagnosed with overt hypothyroidism with positive anti-TPO and anti-Tg autoantibodies that required thyroid hormone substitution therapy; one female patient had been diagnosed with anti-Tg autoantibody-positive subclinical hypothyroidism; one euthyroid female patient was found to have a positive titer of anti-TPO autoantibodies; and one male patient presented subclinical hyperthyroidism with negative anti-TPO, anti-Tg and anti-TSHR autoantibodies. Among these five patients, no evidence for clinical aggravation of TD during or after the DMF treatment was present in the records. In the patient with anti-Tg autoantibody-positive subclinical hypothyroidism, the anti-Tg autoantibody titer decreased from 44.7 kUI/L just before DMF treatment to 25 kUI/l after 4 years of treatment and then to undetectable levels after 6 years of treatment. Conversely, in the euthyroid patient with positive anti-TPO and anti-Tg autoantibodies before DMF treatment, the anti-TPO autoantibody titer increased from 71.0 kUI/l just before DMF treatment to 117.6 kUI/l after 10 months of DMF treatment. In both patients, the elevated autoantibody titers were discovered just before the patients were switched to DMF from interferon beta-1a that they had received during 11 and 13 months, respectively.

**Figure 1:**
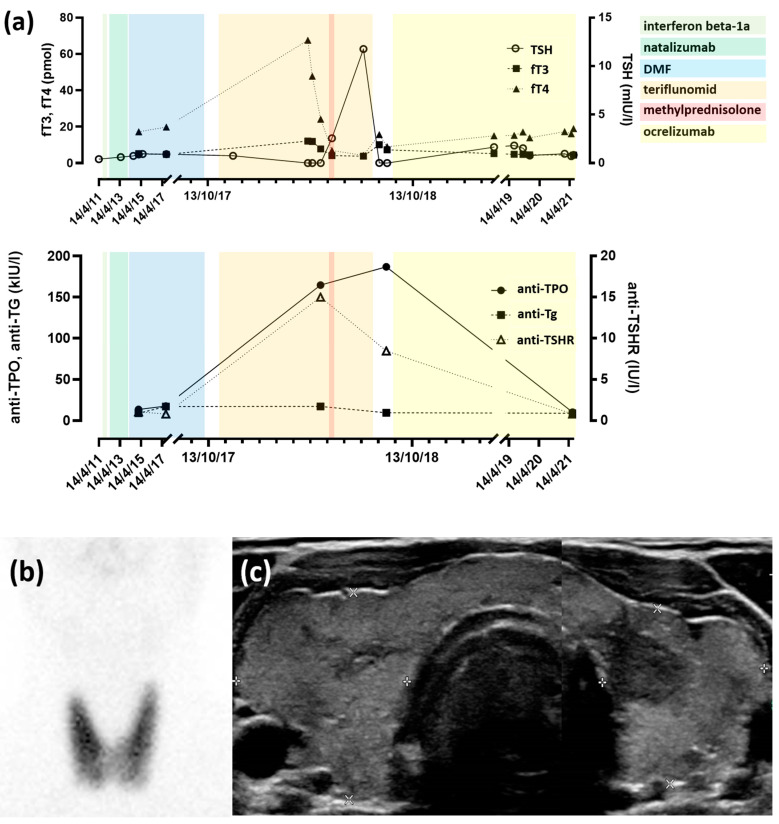
(**a**) Temporal evolution of thyroid function tests and thyroid autoantibody titers in the patient with GD. Treatments were as follows: beta-1a interferon, 44 mcg 3/week (22.06.2011–24.10.2011. We recommend writing in this format, 22 June 2011.); natalizumab, 300 mg 1/month (04.11.2011–06.02.2014); DMF, 240 mg 2/day (11.03.2014–12.10.2017); teriflunomid, 14 mg/day (28.10.2017–20.07.2018); methylprednisone, 1 g/day (02.05.2018–06.05.2018); and ocrelizumab, 300 mg/6 months (since 28.08.2018); (**b**) technetium-99m thyroid scintigraphy showing diffusely increased uptake by the thyroid gland (as compared to very low uptake in the salivary glands); and (**c**) composite ultrasound image of the thyroid gland showing diffuse heterogeneity with alternative isoechoic and hypoechoic areas.

Functional TD were diagnosed during or after DMF treatment in only 2 patients (1.2%). During DMF treatment, one female patient developed mild hypothyroidism with negative anti-TPO, anti-Tg and anti-TSHR autoantibodies; her serum TSH level then normalized spontaneously. Finally, one male patient presented hyperthyroidism after discontinuation of DMF treatment; this was the only patient in the present cohort who presented overt TD and required treatment. In more detail, this was a 40-year-old patient who had been diagnosed with type 1 diabetes in childhood and with MS at the age of 28. After treatment with interferon beta-1a and then natalizumab (an anti-α4β1 integrin monoclonal antibody), he received DMF for 3.5 years, which was discontinued due to persistent lymphopenia (Figure 1). The patient then received treatment with teriflunomid (a dihydroorotate dehydrogenase inhibitor); 6 months after teriflunomid initiation, the patient presented with overt hyperthyroidism with undetectable serum TSH levels and with serum free T4 and free T3 levels at three times and 1.8 times the upper limit of normal, respectively. Graves’ disease (GD) was diagnosed based on a highly elevated titer of anti-TSHR autoantibodies (15.0 UI/L; reference value <1.75 UI/L), a thyroid ultrasound that showed diffuse goiter with increased vascularity, and a thyroid scintigraphy that showed diffusely increased uptake (Figure 1). The patient was rendered euthyroid with anti-thyroid medication within one month, but he could not be weaned and still required treatment at the time of this study, 3 years after the initial diagnosis of GD. Nine months after teriflunomid initiation, the treatment was stopped for lack of efficacy; the patient was next started on ocrelizumab (an anti-CD20 monoclonal antibody) that was ongoing at the time of this study.

### 3.3. Structural TD Is Rare among MS Patients Treated with DMF

Structural TD was found in only 5 (3%) of the total 163 patients treated with DMF. Two female patients were diagnosed with benign non-toxic solitary thyroid nodules, 2 years and 10 years before the introduction of DMF, respectively; in the first case, the nodule was discovered incidentally in the context of a workup for dry mouth. A third female patient was diagnosed with non-toxic multinodular goiter 8 months before DMF treatment that was incidentally discovered in a spine MRI performed for MS follow-up. Furthermore, one male and one female patient were diagnosed with structural TD (a non-toxic solitary nodule and a non-toxic multinodular goiter, respectively) after DMF treatment was discontinued; the former was an incidental finding in a brain MRI performed for MS follow-up. Review of available MRI images revealed that in both patients the nodules were already present 1 and 5 months before the introduction of DMF, respectively. Thus, no patient presented with thyroid structural disease that manifested during or after DMF treatment.

## 4. Discussion

In the present cohorts, the incidence of both structural and functional TD was extremely low during and after treatment with DMF (0% and 1.2%, respectively). In addition, the prevalence of functional TD in our cohorts is comparable with that in the European general population (around 3–4%) [12]; these findings indicate that if thyroidal side effects of DMF exist, they must be rare. Furthermore, we found no evidence that DMF could induce exacerbation of previously known TD; the observed changes in the serum levels of antibodies that target thyroidal antigens in two patients had no impact on thyroid function. These fluctuations of autoantibody titers could be related to the administration and subsequent interruption of interferon beta-1a treatment that the respective patients had before DMF. Indeed, interferon beta-1a is known to induce TD [13] and could be primarily responsible for the elevated titers of anti-TPO and anti-Tg antibodies present before the initiation of DMF.

The single case report describing an acute exacerbation of HT postulated that it was due to the antioxidant effects of DMF rather that its immunomodulatory effects [11]. Indeed, it has been suggested that DMF activates the Keap1/Nrf2 antioxidant response pathway by direct inhibition of the Nrf2-Keap1 interaction via reaction with cysteine residues of Keap1, as well as indirectly, following an initial depletion of reduced glutathione (GSH) [14] that provokes transient oxidative stress [15]. Oxidative damage resulting from oxidative stress could trigger the development of immunological intolerance in HT [16]. In our cohort, only one patient developed AIT, specifically, GD. As with other treatments for MS, modulation of the immune system precipitating GD could be involved. Indeed, DMF is believed to act in MS by Nrf2-independent neuroprotective mechanisms as well as through modification of the composition, phenotype and CNS migration of immune cells [3,17]. Alemtuzumab, an anti-CD52 monoclonal antibody used as a treatment for MS, frequently induces AIT (in 20% to 33% of patients), probably due to rapid recovery of CD8 T-lymphocytes after alemtuzumab-induced lymphocytes depletion [18]. Similar mechanisms in response to DMF could be postulated as causal for GD in the present patient, but it cannot be excluded that GD was triggered by the subsequent teriflunomid treatment. Although TD are not usually associated with teriflunomid, a 41-year old woman with MS and an autoimmune polyglandular syndrome was reported to develop transient hypothyroidism during teriflunomid treatment [19].

Even if DMF might exacerbate AIT in rare cases through the aforementioned mechanisms or others undescribed until now, there is also evidence that supports that DMF could be protective against chronic diseases, notably autoimmune disorders (AID) [20]. Regarding the thyroid gland more specifically, SNPs in the gene encoding Nrf2, including a SNP known to impact promoter activity, have been shown to modulate the risk of autoimmune thyroiditis in conjunction with SNPs in the gene encoding selenoprotein S; alleles associated with higher Nrf2 promoter activity were protective in that context [10]. Furthermore, Nrf2 is known to directly upregulate the transcription of the gene encoding Tg but to downregulate Tg iodination. The inhibition of Tg iodination could prevent the development of autoimmunity, because it is known that enhanced and aberrant iodination of Tg promotes autoimmune reactivity in the thyroid [21]. Finally, in a retrospective study nested in a 12-week randomized trial conducted in China that tested sulforaphane, a natural activator of Nrf2, for the detoxification of airborne pollutants, we found no effects on the thyroid hormonal profile or the anti-TPO and anti-Tg auto-antibody status [22]. These lines of evidence suggest that pharmacological activation of Nrf2 is likely safe for the thyroid and might even be beneficial to protect against oxidative stress-related pathologies such as AIT.

More generally, it is important to note that DMF is a relatively unspecific Nrf2 activator and has many other cellular targets, in addition to Keap1, the main negative regulator of Nrf2. For example, DMF has been shown to bind and inactivate the catalytic cysteine of the glycolytic enzyme glyceraldehyde 3-phosphate dehydrogenase (GAPDH). Consequently, DMF down-regulates aerobic glycolysis in activated myeloid and lymphoid cells, which has anti-inflammatory effects [23]. It is also worth mentioning that Nrf2 is not the only target of Keap1, and inhibition of Keap1 by DMF can promote mitochondrial-targeted apoptosis of certain immune cells, such as neutrophils and macrophages by causing dissociation of WD repeat domain 1 (Wdr1) from Keap1 and subsequent coordination with cofilin, as shown recently [24].

This work has some limitations. First, it is a retrospective observational study in one cohort where thyroid function tests and autoantibody titers were not systematically recorded according to a standardized protocol. The proportion of patients with at least one TSH value before, during and, if applicable, after DMF treatment was relatively low (18%), and we had access only to results of test performed in our hospital or archived from external reports. Nevertheless, even though the prevalence of abnormal thyroidal test parameters in our cohorts may be underestimated, the incidence of clinically relevant TD associated with DMF treatment is likely much less affected.

In mice, genetic activation of Nrf2 signaling secondary to decreased expression of Keap1 causes age-dependent subclinical hypothyroidism and goiter [25]. In our cohorts, thyroid imaging was not systematically performed in the context of DMF treatment. Indeed, the U.S. Preventive Service Task Force and others [26,27,28] currently recommend against thyroid screening for thyroid nodules in absence of specific risk factors. Therefore, the prevalence of structural TD in our cohorts is most certainly underestimated. Nevertheless, as for functional TD, the incidence of clinically relevant goiter associated with DMF treatment is likely much less affected.

Finally, we did not compare the prevalence of thyroid disorders with a control group of patients with MS who did not receive DMF treatment. The reason is that patients who did receive DMF were treated with other immunomodulatory treatments for MS, and such drugs, including interferon beta-1a [13], alemtuzumab [29], and ocrelizumab [30] have already been described to induce thyroid gland dysfunction, thus precluding the use of those patients as controls.

## 5. Conclusions

The present study suggests that DMF probably has no direct toxic effect on the thyroid, as evidenced by a very low incidence of functional TD; however, it cannot be excluded that in rare instances it might induce AIT in susceptible patients with or without known preexisting TD. The overall good thyroid tolerance of DMF indicates that specific screening for TD is not required in MS patients considered for or treated with DMF. Finally, it supports the further study of this molecule and other activators of the Nrf2/Keap1 antioxidant response for possible utility in the prevention and treatment of TD.

## Data Availability

All data supporting reported results are provided as Supplementary Materials.

## Supplementary Materials

The following supporting information can be downloaded at: https://www.mdpi.com/article/10.3390/antiox11051015/s1, Table S1: Raw patient data used in this study. The following parameters are included: (1) Arbitrary patient number. (2) Gender; female (F) or male (M). (3) Age at DMF introduction (in of patients who received more than one courses of DMF, the age corresponds to the start of the first course). (4) “DM start”; date of DMF introduction. (5) “DM end”; date of DMF termination. The table also indicates if at least one TSH value was either in the reference range (normal, “*n*”) or below or above the reference range (abnormal, “ab”) before (6), during (7) or after (8) DMF treatment. In patients still receiving DMF treatment at the end of the present study, TSH value after DMF treatment is not applicable (“na”). (9) “dysthyroidism”; indicates the presence (“yes”) or absence (“no”) of thyroid gland disorders based on TSH values or the mention of thyroid gland disorders in the patients record. (10) “thyroid diagnosis” indicates specific thyroid gland disorders, whenever present. The following columns of Supplementary Table S1 describe the different multiple sclerosis-specific treatments that the patients received: treatment name (columns 11, 19, 27, 35, 43, 51, 59 and 67); treatment start date (columns 12, 20, 28, 36, 44, 52, 60 and 68); treatment end date (columns 13, 21, 29, 37, 45, 53, 61 and 69); dosage (columns 14, 22, 30, 38, 46, 54, 62 and 70); units (columns 15, 23, 31, 39, 47, 55, 63 and 71); frequency (columns 16, 24, 32, 40, 48, 56, 64 and 72); route of administration (columns 17, 25, 33, 41, 49, 57, 65 and 73); and, in case of discontinuation (columns 18, 26, 34, 42, 50, 58, 66 and 74), the specific reason, if known. Tables S2–S8 present the thyroid test results (TSH, total T3, free T3, total T4, free T4, anti-TPO, anti-Tg and anti-TSHR, whenever available) of the respective patients with abnormal thyroid test values. Table S2: Data of patient 9; Table S3: data of patient 15; Table S4: data of patient 62; Table S5: data of patient 97; Table S6: data of patient 103; Table S7: data of patient 121; Table S8: data of patient 153.
